# A water quality assessment method based on an improved grey relational analysis and particle swarm optimization multi-classification support vector machine

**DOI:** 10.3389/fpls.2023.1099668

**Published:** 2023-01-25

**Authors:** Rongli Gai, Zhibin Guo

**Affiliations:** School of Information Engineering, Dalian University, Dalian, China

**Keywords:** grey relational analysis, particle swarm optimization, support vector machine, water quality assessment, feature selection

## Abstract

Most of the water quality indicators that affect the results of river water quality assessment are gray and localized, thus the correlation between water quality indicators can be calculated using gray correlation analysis (GRA).However, GRA takes equal weighting for water quality indicators and does not take into account the weighting of the indicators. Therefore, this paper proposes a river water quality assessment method based on improved grey correlation analysis (ACGRA) andparticle swarm optimization multi-classification support vector machine (PSO-MSVM) for assessing river water environment quality. Firstly, the combination weights of water quality indicators were calculated using Analytic Hierarchy Process (AHP)AHP and Criteria Importance Though Intercrieria Correlation (CRITIC)CRITIC, and then the correlation between water quality indicators was calculated for feature selection. Secondly, the PSO-MSVM model was established using the water quality indicators obtained by ACGRA as input parameters for water environment quality assessment. The river water environment assessment methods of ACGRA and PSO-MSVM were applied to the evaluation of water environment quality in different watersheds in the country. Accuracy, precision, recall and root mean square errorRMSE were also introduced as model evaluation criteria. The results show that the river water environment assessment methods based on ACGRA and PSO-MSVM can evaluate the water environment quality more accurately.

## 1 Introduction

China is rich in river water resources, and is an important water source for living, industry and agriculture in coastal areas. Protecting river water environment security is of great significance to the economic and social development and people’s life in coastal areas of river basins (Yan et al., 2019). River water quality influenced by many water quality index factors such as dissolved oxygen, temperature, turbidity, ammonia nitrogen, and some other factors. If we can fully use water quality index factors, it will help improve the accuracy of water environmental quality assessment. Since river water quality is influenced by many water quality index factors, it will help to improve the accuracy of water environment quality assessment if we can make full use of these water quality index factors.

With the rapid development of machine learning (ML), ML-based water quality assessment methods have become a hot research topic (Zheng et al., 2022). For example, Zhao, Z et al. proposed a water quality assessment model based on a stochastic hybrid dynamic system (Zhao et al., 2022). In addition, Gao, Z et al. presented an enhanced beetle antenna search algorithm for urban river water quality assessment (Gao et al., 2022). Further, Tang, M et al. segmented the water quality assessed by the water quality deterioration rate index Likewise (Tang et al., 2022), Chopade, S et al. combined deep neural networks with sensors to build a river water quality assessment system (Chopade et al., 2021). The above methods consider the influence of multiple indicators on the quality of the water environment in the assessment. However, these methods do not analyze the correlation between water quality indicators.

There are variable and complex correlations between water quality indicators (e.g., non-linear correlations between water quality grades and multiple indicators such as dissolved oxygen, temperature, turbidity, and permanganate index). Therefore, we need to calculate the correlation between different indicators, from which we can select the appropriate characteristics for water quality assessment. Commonly used correlation analysis methods include Pearson correlation coefficient analysis (Zhang et al., 2022a), Granger causality analysis (Tang et al., 2020), and grey relational analysis (Wang et al., 2020). Most indicators affecting water quality are grey and localized. Thus, GRA is well suited to solve this type of problem (Zhou et al., 2018). However, GRA uses equal weighting in calculating the correlation of water quality indicators and does not consider the weight of the indicators. To this end, this paper proposed an improved grey relational analysis algorithm (ACGRA) to measure the correlation between indicators more accurately and then use it to perform feature selection from the indicators.

SVM has excellent advantages in dealing with small samples and complex nonlinear model problems. Compared with other algorithms, it has advantages such as fast learning speed and strong generalization ability (Tan et al., 2019). Therefore, a support vector machine (SVM) can be used for classification of features obtained by the ACGRA as input parameters. However, the selection of parameters has a significant impact on the performance of the model. In order to obtain accurate evaluation results, it is necessary to obtain the best parameters. Traditional parameter selection methods include K-fold cross-validation (Jung et al., 2020), grid search (Dinh and Van der Baan, 2019), and genetic algorithm (Duvvuri and Anmala, 2019). However, these methods are time-consuming and low in efficiency. In order to solve this problem, particle swarm optimization has the characteristics of global optimization, and the particles have memory. Therefore, this paper uses a particle swarm optimization algorithm to optimize SVM parameters and further improve water quality assessment accuracy.

In order to make full use of the correlation between water quality indicators and accurately evaluate the river water quality. In this paper, a water quality evaluation method based on the combination of ACGRA and PSO-MSVM is proposed. First, ACGRA was used for feature selection of water quality indicators. Then, a water quality assessment model was developed using PSO-MSVM, whose inputs were the indicators obtained from ACGRA. The method was compared with other similar methods in the Chinese river cross-sectional water quality monitoring dataset for January-December 2021. The details of the evaluation can be seen in the next sections, and the method proposed in this paper outperforms other similar methods in assessing water quality. The method overcomes the problem that the GRA algorithm does not consider the importance of indicators when calculating the correlation of indicators, and improves the accuracy of river water quality evaluation, which is very helpful for the intelligent management of water environment.

The most important contributions of this article are as follows:

(1) A new technique called ACGRA method is used for computing correlations between water quality indicators for feature selection.
(2) Optimization of multi-classification support vector machine parameters using particle swarm algorithm to improve the performance of water quality assessment models.

The remaining parts of the paper are structured as follows: Section 2 describes the study area and the dataset used for the experiments. In addition, water quality assessment methods based on ACGRA and PSO-MSVM are provided in Section 3. Further, Section 4 presents model-related evaluation metrics and provides an experimental comparative analysis with other methods. The full text is summarized in Section 5.

## 2 Study area and dataset

### 2.1 Study area

**Figure 1:**
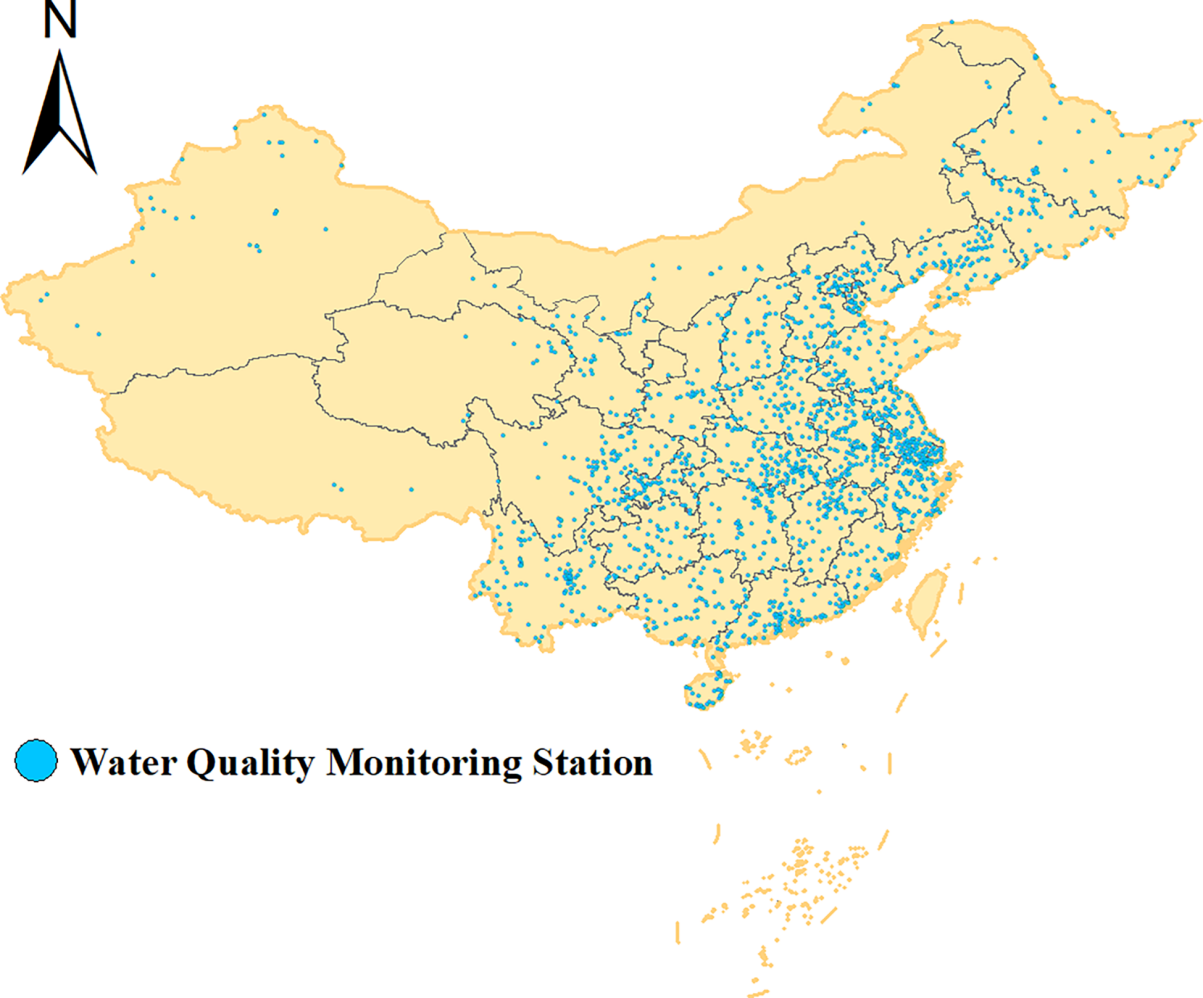
Distribution of water quality monitoring stations.

This study is based on the national river cross-sectional water quality monitoring dataset, the location of water quality monitoring points (**Figure 1**), where the Yangtze River and Huaihe River basins are the most abundant river water resources, and the basin area of about 1.27 million square kilometers. It is also based on the river flows through Henan, Jiangsu, Hubei, Zhejiang, Hunan, Fujian, and other provinces and autonomous regions, affecting more than 250 million residents. Therefore, it isof great strategic importance to monitor the water quality of domestic rivers and protect the water quality safety of rivers. At the same time, the data and information obtained from monitoring can also provide a scientific basis for pollution source control, environmental planning, etc.

### 2.2 Dataset

The dataset used in this paper is the monthly data of water quality monitoring at each river cross-section nationwide from January to December 2021 (**Table 1**). The monitoring dataset consists of seven parameter features, including dissolved oxygen, temperature, turbidity, ammonia nitrogen, permanganate index, ph, and total dissolved solid.

**Table 1:** River water quality data characteristics.

Parameter	Unit	Minimum	Maximum	Average
DO	mg/L	0.5	25.67	9.03
TEMP	°C	0.04	35.22	16.85
Turbidity	NTU	0.22	2559.22	47.03
NH_3_-N	mg/L	0.02	6.47	0.19
CODmn	mg/L	0.25	22.66	3.20
PH	mol/L	6.14	9.98	1.63
TDS	mol/L	47.43	323.12	185.07

DO, Dissolved oxygen; TEMP, Temperature; NH_3_-N, Ammonia nitrogen; CODMn, Permanganate index; TDS, Total dissolved solid.

According to China’s Surface Water Environmental Quality Standard (GB3838-2002), water quality in the dataset was divided into six different categories, including I, II, III, IV, V, and poor V (**Table 2**). Considering that there are no standard limits forturbidity and total dissolved solid of river cross-section water quality, the standard limits for turbidity and total dissolved solid of drinking water were used in the evaluation process. Water quality responds to the impact of industry, agriculture, and other sources of river water pollution along the river on water quality.

**Table 2:** Environmental quality standard limits for surface WaterRiver water quality data characteristics.

	Category				
Parameter	I	II	III	IV	V
DO	7.5	6	5	3	1
TEMP (°C)	Maximum weekly average temperature rise ≤ 1, maximum weekly average temperature drop ≤ 2				
Turbidity(NTU) ≤	1NTU				
NH_3_-N ≤	0.15	0.5	1.0	1.5	2.0
CODmn ≤	2	4	6	10	15
PH	6-9				
TDS ≤	150	300	450	550	550

DO, Dissolved oxygen; TEMP, Temperature; NH_3_-N, Ammonia nitrogen; CODMn, Permanganate index; TDS, Total dissolved solid.

## 3 Proposed methods

### 3.1 Feature selection based on ACGRA

GRA is a method for assessing the degree of interaction between indicators through geometric similarity between them (Zhang et al., 2021), and it takes a leveling treatment of the indicators. Sutadian et al. proposed a subjectively empowered method for calculating the correlation of indicators (Sasikumar et al., 2022). Miao, C et al. also introduced an objectively assigned indicator correlation calculation (Xu et al., 2020). However, their method only considers the influence of a single factor on the correlation of indicators rather than considering subjective and objective factors together. Hence, the results often do not accurately reflect the correlation between indicators. To compensate for this deficiency, the ACGRA method was proposed in this paper. The calculation process and equation are as follows:

Set the parent indicator *X*_0_(*i*)={*x*_0_(1),*x*_0_(2),…,*x*_0_(*i*)} , where *X*_0_(*i*) denotes the water quality category of the first *i* water quality data of *X*_0_, and *x*_0_ (*i*) is for the water quality category of the *i* water quality data. Then set the subindex *X*_*j*_(*i*)={*x*_*j*_(1),*x*_*j*_(2),…,*x*_*j*_(*i*)} , where *X*_*j*_(*i*) represents the first *i* water quality data of *X_j_*, and *x_j_* (*i*) indicates the *i* water quality data of *X_j_*. Set the parent indicator *X*_0_(*i*)={*x*_0_(1),*x*_0_(2),…,*x*_0_(*i*)} , where *X*_0_(*i*) denotes the water quality category of the first *i* water quality data of *X*_0_, and *x*_0_(*i*) is for the water quality category of the *i* water quality data. Then, set the subindex *X*_*j*_(*i*)={*x*_*j*_(1),*x*_*j*_(2),…,*x*_*j*_(*i*)} , where *X j* (*i*) represents the first *i* water quality data of *X_j_*, and *x_j_* (*i*) indicates the *i* water quality data of *X_j_*. Thedata are normalized, where *X_j_* (*i*) is the normalized value of the subindex.


(1) $$X_{j}{\left(i\right)}^{'}=\frac{X_{j}\left(i\right)-min\;\left(X_{j}\right)}{max\;\left(X_{j}\right)-min\;\left(X_{j}\right)},i=1,2,\ldots ,m;j=1,2,\ldots ,n$$

Determine the number of grey-off contacts, where s*_j_* (*i*) is the PSO-MSVM coefficient between the subindex and the parent index. In addition, Δ_*max* _ and Δ_*min* _ are the maximum and minimum values of *X*_0_(*i*) and *X*_*j*_(*i*)^'^ absolute difference, respectively, and *ρ* is the difference coefficient, which is usually set to 0.5 (Zavareh et al., 2021).


(2) $$s_{j}\left(i\right)=\frac{\Delta_{min\;}+\rho \Delta_{max\;}}{\Delta_{j(i)}+\Delta_{max\;}}$$

Calculate weight. First, the hierarchical structure was constructed for the research problem, after which the comparison matrix *Q*=(*q*_*jl*_)_*n*×*n*_ was obtained using the 1-9 level scaling method (Coffey and Claudio, 2021), and then the comparison matrix was calculated using Eq. (3) to obtain subjective weights *α*_*j*_ as follows:


(3) $$\alpha_{j}=\sum_{l=1}^{n}\frac{q_{jl}}{\sum_{j=1}^{n}q_{jl}},\left(j,l=1,2,\ldots ,n\right)$$

The amount of information *C*_*j*_ for each attribute can be computed using Eq. (4), where *σ*_*j*_ denotes the standard deviation of each indicator, and *r*_*ij*_ is the correlation coefficient of the *i* indicator data with the jcategory.


(4) $$C_{j}=\sigma_{j}\sum_{i=1}^{m}\left(1-r_{ij}\right)$$

The objective weight *β*_*j*_ of each attribute can be determined by Eq. (5) as:


(5) $$\beta_{j}=\frac{C_{j}}{\sum_{j=1}^{n}C_{j}}$$

Eq. (6) was used to calculate the combination weights *ω*_*j*_


(6) $$\omega_{j}=\left(\alpha_{j}+\beta_{j}\right)/\sum_{j=1}^{n}\left(\alpha_{j}+\beta_{j}\right)$$

Eq. (7) was employed to compute the index correlation *r*(*j*) .


(7) $$r\left(j\right)=\frac{1}{m}\sum_{i=1}^{m}\left(s_{j}\left(i\right)\times \omega_{j}\right)$$

### 3.2 Water quality assessment based on PSO-MSVM

An SVM is a binary classifier that can only distinguish between two different water quality categories. There are currently two methods for SVMs to solve multi-classification problems, including one-to-over (OVR) (Abe, 2015) and one-to-one (OVO) (El Mamoun et al., 2019). OVR is the synthesis of multiple categorical solution problems into a single optimization solution problem that seeks to be solved in one go. However, it is not often used due to its high computational complexity, difficulty in implementation, and low accuracy. In this paper, OVO was applied to solve the multi-classification problem. It is to design a hyperplane between any two classes of samples to construct $\frac{k\left(k-1\right)}{2}$ classifiers. When classifying the unknown samples, the classifier takes a vote, and the final category with the most votes is the category of the unknown samples.

The choice of SVM parameters and the quality of the kernel function have a great impact on the performance of the SVM model (Haixiang et al., 2017). **Table 3** provides the commonly used kernel functions and their corresponding parameters. Given that the kernel function chosen in this paper is the radial basis kernel function, the optimal values of parameters *C* and *r* should be considered to obtain accurate evaluation results. In this paper, we use particle swarm algorithm to optimize SVM parameters, so as to further improve its water quality assessment accuracy.

**Table 3:** Kernel function formula and parameters.

Kernel function	Formula	Parameters
Linear	(*x*·*y*)	*C*
Rbf	*exp* (−||*x*−*y*||^2^/*r* ^2^)	*C*, *r*
Polynomial	((*x*,*y*)+1)^*d*^	*C*, *d*

**Figure 2:**
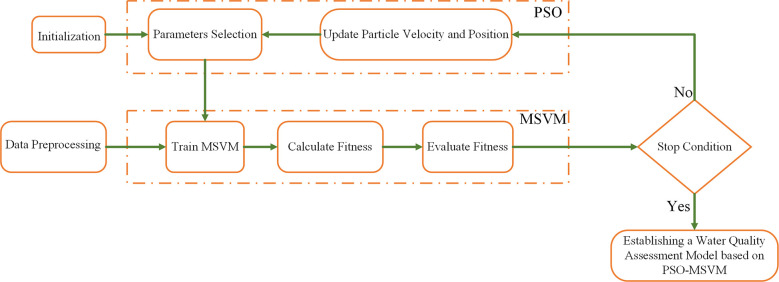
Flowchart of PSO-MSVM parameter optimization.

PSO is a population-based intelligent optimization stochastic search algorithm (Lu et al., 2022). First, the initialized particle swarm is generated as a solution in the feasible space. Then, the search iterations continue among the particles to continuously modify the feasible solutions and finally find the global optimal solution *G*_*bt*_ (Hameed et al., 2017). The particles will be updated in each search iteration by the local optimal solution *P*_*bt*_ and the global optimal solution *G*_*bt*_ (**Figure 2**). The calculation steps are as follows:

First, set the initial value of the particle swarm and the maximum number of iterations *T*_*max*_. Assuming that the particle swarm is iteratively searched in an n-dimensional space, the position of the *j* particle is denoted by *Z*_*j*_={*z*_*j*1_,*z*_*j*2_,…,*z*_*jm*_} , and the velocity of the *j* particle is represented by *V*_*j*_={*v*_*j*1_,*v*_*j*2_,…,*v*_*jm*_} .

Second, after finding *P*_*bt*_ and *G*_*bt*_ , the velocity of the *j* particle is updated by the following two equations:


(8) $$V_{j}^{t+1}=\omega V_{j-1}^{t}+c_{1}r_{1}\left(P_{bt}-Z_{j}\right)+c_{2}r_{2}\left(G_{bt}-Z_{j}\right)$$

(9) $$Z_{j}^{t+1}=Z_{j}^{t}+V_{j}^{t+1}$$

where *ω* is the inertia weight. Further, *c*_1_ and *c*_2_, as well as *r*_1_ and *r*_2_ are learning factors and arbitrary numbers in the range of [0,1], respectively.

The third section is related to iterative search termination conditions. There are two conditions for terminating the iteration; the first one is that the precision is less than the set error value and the particle swarm converges, thus the iteration can be stopped, and the second one is that the number of iterations reaches the set maximum number of iterations *T*_*max*_ .

Finally, the optimal parameters obtained after the iterations are used to build a water quality assessment model.

When *c*_1_ is larger than *c*_2_, it leads to a part of particles being trapped in the local search, eventually leading to the inability to find the global optimal solution. Therefore, the value of *c*_1_ is taken as 1.6, and the value of *c*_2_ is taken as 2 in the model of this paper. Although when *ω* is larger, the global search capability is stronger, the algorithm is extremely prone to fall into local extremes when *ω*>1.2. Thus, to enable the particles to detect globally better regions in the early stage, while ensuring the convergence of the algorithm to the global optimal solution in the later stage, a linear decreasing weight strategy was chosen with the following equation:


(10) $$\omega =0.9-0.4*\frac{t}{T_{max\;}}$$

where *t* represents the number of current iterations.

### 3.3 Water quality assessment method based on ACGRA and PSO-MSVM

**Figure 3:**
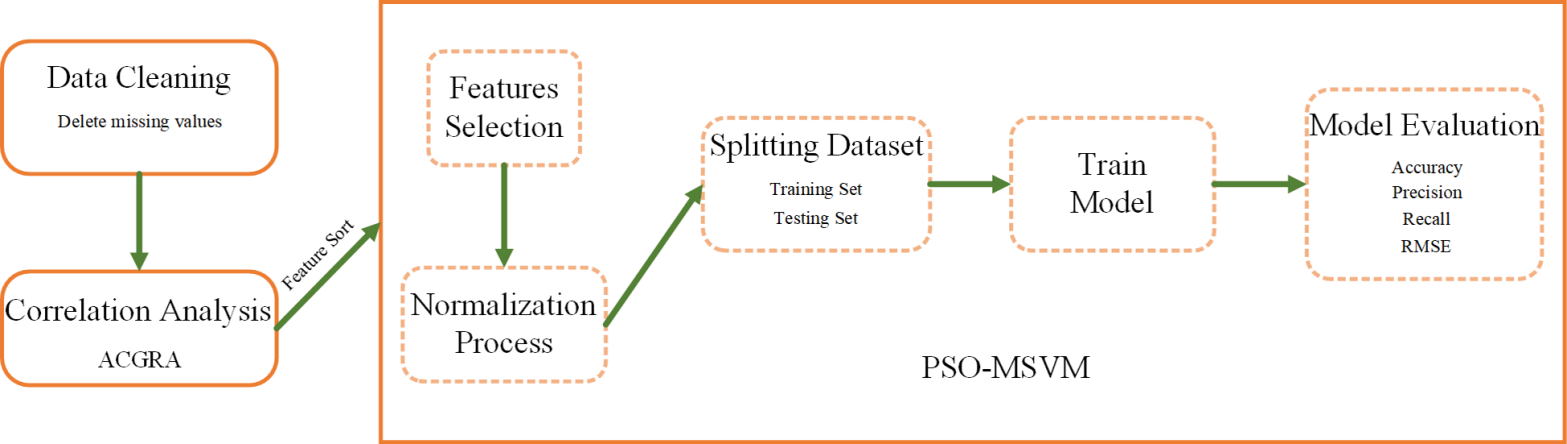
Flowchart of the river water quality assessment method based on ACGRA and PSO-MSVM.

The flow of the water quality assessment method based on ACGRA and PSO-MSVM is shown in **Figure 3**. To make full use of the correlation between water quality information, the features were selected from the water quality information using the method in Section 3.1, and the water quality assessment model was built using the method in Section 3.2.

The specific steps of the river water quality assessment are as follows:

Step 1: Cleaning the data and normalizing the dataset;
Step 2: Using ACRGA to calculate the correlation coefficients between water quality indicators and categories and selecting indicators with large correlation coefficients as characteristics;
Step 3: Normalizing the data;
Step 4: Dividing the training and test sets and building the PSO-MSVM-based water quality assessment model and then training by the training set;
Step 5: Evaluating the test set water quality data to calculate the accuracy, precision, recall, and root mean squared error (RMSE) of the model.

## 4 Experiments and results

### 4.1 Related evaluation indicators

Accuracy, precision, recall, and RMSE criteria were used to evaluate the performance of the model. Accuracy is defined as:


(11) $$\text{ Accuracy }=\frac{TP+TN}{TP+TN+FP+FN}$$

Precision is defined as:


(12) $$\text{ Precision }=\frac{TP}{TP+FP}$$

Recall is defined as:


(13) $$\text{ Recall }=\frac{TP}{TP+FN}$$

where *TP*, *TN*, *FP*, and *FN* represent the true case, false positive case, true negative case, and false negative case, respectively.

RMSE is defined as:


(14) $$\text{ RMSE }=\sqrt{\frac{1}{k}\sum_{i=1}^{k}{\left(Y_{i}^{'}-Y_{i}\right)}^{2}}$$

where *k* is the number of samples, *Y_i_* and $Y_{i}^{'}$ are the actual and evaluated values of the samples, respectively.

### 4.2 Feature selection results

In this paper, different correlation analysis methods were employed to calculate the direct correlation between water quality and other water quality indicators, and the results are presented in **Table 4**.

**Table 4:** Correlation of water quality indicators.

	DO	TEMP	Turbidity	NH_3_-N	CODmn	PH	TDS
Literature (Sasikumar et al., 2022)	0.1918	0.1845	0.1876	0.0918	0.1739	0.0954	0.0282
Literature (Xu et al., 2020)	0.0278	0.6387	0.0017	0.0021	0.0339	0.1264	0.1154
ACGRA	0.1098	0.4116	0.0946	0.0470	0.1039	0.1109	0.0718

DO, Dissolved oxygen; TEMP, Temperature; NH_3_-N, Ammonia nitrogen; CODMn, Permanganate index; TDS, Total dissolved solid.

Based on data in **Table 4**, ACGRA fully considers the effect of subjective and objective weights on the correlation between water quality and other water quality indicators compared to the single weighted grey correlation analysis used in the literature literature (Sasikumar et al., 2022) and literature (Xu et al., 2020).

To further verify the effectiveness of ACGRA, four water quality indicators with high correlation with water quality (**Table 4**) were selected as the input features of the PSO-MSVM-based water quality assessment model, and the model errors are provided in **Table 5**.

**Table 5:** Feature selection and model error.

	Feature	RMSE
Literature (Zhang et al., 2022b)	NH_3_-N,CODmn,PH,TDS	0.5527
Literature (Sasikumar et al., 2022)	DO,TEMP,NH_3_-N,CODmn	0.3593
Literature (Xu et al., 2020)	TEMP,CODmn,PH,TDS	0.4367
ACGRA	DO,TEMP,CODmn,PH	0.3322

DO, Dissolved oxygen; TEMP, Temperature; NH_3_-N, Ammonia nitrogen; CODMn, Permanganate index; TDS, Total dissolved solid.

The results of water quality assessment using features obtained by weighted grey relational analysis are better compared to previous research literature (Zhang et al., 2022b) using features obtained by equal-weighted grey relational analysis for water quality assessment (**Table 5**). Compared to the improved GRA algorithm in studies by literature (Sasikumar et al., 2022) and literature (Xu et al., 2020), the RMSE is smaller when selecting the characteristic variables with ACGRA. This indicates that ACGRA can make full use of the correlation between water quality indicators and can effectively improve the accuracy of river water quality assessment.

### 4.3 Kernel selection results

**Figure 4:**
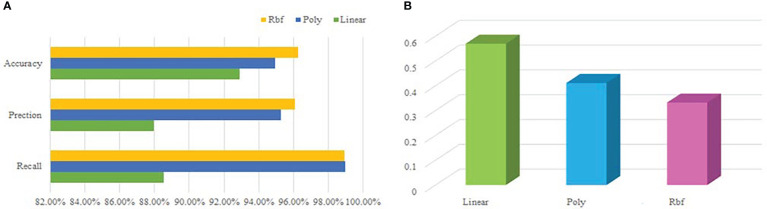
Results of different kernel function choices **(A)** Accuracy, precision and recall of model **(B)** RMSE for model.

In this paper, radial basis function (Rbf) is selected as the kernel function of SVM model. In order to verify the effectiveness of the Rbf, it is compared with the Linear Kernel (Linear) and Polynomial Kernel (Poly), and the results are shown in **Figure 4**.

As can be seen from **Figure 4A**, when the kernel function is Rbf, the accuracy, accuracy and recall rate of the model are all higher than those of the other two types of kernel functions, and the RMSE of the three types of kernel functions is shown in **Figure 4B**. The results show that the model can evaluate water quality more accurately when radial basis function is selected as kernel function.

### 4.4 Algorithm optimization results

The choice of SVM parameters had a great influence on the effectiveness of water quality assessment models, thus the particle swarm algorithm was used to optimize the SVM so as to obtain the best parameters.

**Figure 5:**
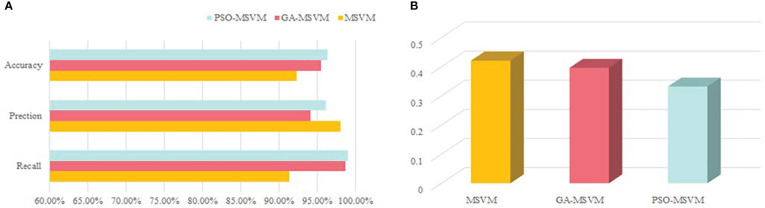
Results of different optimization algorithms. **(A)** Accuracy, precision and recall of model. **(B)** RMSE for model.

To verify the effectiveness of the PSO algorithm, it was compared with the multi-classification SVM model optimized by the multi-classification SVM and genetic algorithm (**Figure 5**).

As shown in **Figure 5A**, the multi-classification SVM model using PSO has higher accuracy, precision, and recall and lower RMSE (**Figure 5B**). Based on the results, the particle swarm algorithm could better optimize the multi-classification SVM algorithm and effectively improved the performance of the water quality assessment model.

### 4.5 Water quality assessment results

In this paper, the initial particle population and the maximum number of iterations were set to 300 and 300, respectively, and the learning factors *c*_1_ =1.6 and *c*_2 =_ 2._ A_ river water quality assessment model based on PSO-MSVM was developed using water quality indicators such as dissolved oxygen, temperature, turbidity, ammonia nitrogen, permanganate index, ph, and total dissolved solid of the river.

**Figure 6:**
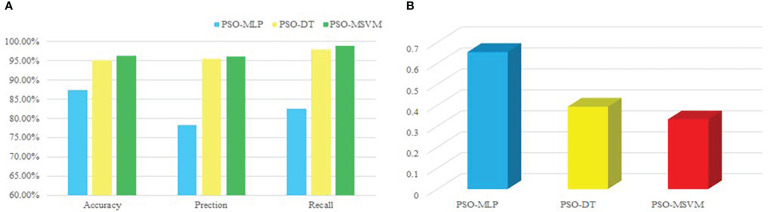
PSO-DT, PSO-MLP, PSO-MSVM model performance values. **(A)** Accuracy, precision and recall of model. **(B)** RMSE for model.

To verify the effectiveness of PSO-MSVM, it is compared with the PSO-DT and PSO-MLP algorithm models, and the results are illustrated in **Figure 6**.

Based on data in **Figure 6A**, the PSO-MSVM model has higher accuracy, precision, and recall compared to the other two algorithms, and the RMSEs of the three methods are displayed in **Figure 6B**. The results demonstrate that the PSO-MSVM model is more accurate in assessing river water quality.

## 5 Conclusion

River water quality assessment is important for water environmental protection. Considering the weight and relevance of water quality information, a water quality assessment method based on ACGRA and PSO-MSVM was proposed in this study. First, the correlation of water quality information was calculated using ACGRA, and the four indicators with the greatest correlation were selected as input features. Then, the characteristics obtained by ACGRA were input into the PSO-MSVM model to assess the quality of the river water environment. This method was compared with other similar methods in the water quality monitoring data set of river sections in China from January to December 2021.

The experimental results show that ACGRA can effectively solve the problem of GRA equilibrium calculation, make full use of the weight and correlation of water quality indexes, and effectively select the main influencing indexes for water quality evaluation. And PSO-MSVM model can effectively overcome the problem of system parameter selection in support vector machine, make full use of water quality information, so as to improve the evaluation accuracy. However, using ACGRA for feature selection requires adequate water quality indicators. In addition, the large number of iterations of the particle swarm algorithm results in a long training time for the model. Further, given that the multi-classification SVM uses a voting mechanism to determine the result, the number of classifiers is required to be odd. In the future, the use of graphic processing units (GPUs) for training or improved particle swarm algorithms can be considered to increase the speed of the model and improve the multi-classification SVM algorithm so that it is also employed for the case of an even number of classifiers. The use of GPU for training can be considered in the future. The method can also be applied to the water quality assessment of drinking water, lakes, and oceans. Finally, in the future, it can be extended to air quality assessment, personal reputation assessment, and the like.

## Data availability statement

The raw data supporting the conclusions of this article will be made available by the authors, without undue reservation.

## Author contributions

All authors designed the experiment and wrote the manuscript. All authors contributed to the article and approved the submitted version.
